# Evaluating biometrics by using a hybrid MCDM model

**DOI:** 10.1038/s41598-021-00180-2

**Published:** 2021-10-21

**Authors:** Hung-Jia Tsuei, Guiping Shen, Gwo-Hshiung Tzeng

**Affiliations:** 1 College of Artificial Intelligence, Yango University, Fuzhou, 350015 Fujian China; 2 Graduate Institute of Urban Planning, National Taipei University, New Taipei City, 23741 Taiwan

**Keywords:** Biotechnology, Computational biology and bioinformatics, Mathematics and computing

## Abstract

Biometrics has been developing for decades in diverse industries, such as consumer electronics, internet of things, financial industry, etc. The purpose of this research is to build a decision-making model to evaluate and improve the performances of biometrics for administrators to design and make suitable biometric systems. This paper adopts a hybrid multiple criteria decision making (MCDM) model, comprising decision-making trial and evaluation laboratory (DEMATEL), and DEMATEL-based analytic network process (called DANP) to probe into the interrelationship and influential weights among criteria of biometrics. According to DEMATEL technique, the empirical results indicate that criteria of biometrics have self-effect relationships. The dimension of biometrics that administrators of biometrics should enhance first when improving the performances is usability. The criterion of universality with the highest influencing value to systematically affect all other evaluation factors is what administrators of biometrics should comprehensively consider. In the top three criteria for evaluation by DANP, biometric systems with the most influential weight is the criterion that can be improved to have higher recognition rates for increasing the performances of biometrics, followed by biometric conditions and permanence.

## Introduction

Biometrics are the science and technology of measurement and statistical analysis on the information of physiological characteristics and behavioral characteristics. Biometrics for human recognition are widely used by both the public and private institutions, and thus the greater security and improved service are highly stressed^1,2^. Furthermore, four distinctive traits have been discovered: physiological, behavioral, medico-chemical such as Deoxyribonucleic Acid (DNA) and Electrocardiogram (ECG), and soft such as height, gender, ethnicity^3,4^.

Biometric authentication systems play an important role in recent years in the fields of commercial electronics, security, military, finance, etc^5^. Besides, the developments of bioelectronics have in turn impacted on biometrics, such as the DNA identifier. In addition, prompt advancements in communication technologies and expansion of consumer electronic devices have brought novel applications for biometrics^6,7^. Moreover, there is no biometric technology with one hundred percent safe, because direct (spoofing) or indirect attacks and the strength of privacy preservation techniques make the reliability of biometric systems unknown. The reliability of biometric technologies cannot be estimated for the deficiency of standard evaluation models^8–10^. In addition, the multiple criteria decision making (MCDM) model was used to evaluate the usability for biometric system, because the elements of biometrics were critical for usability and security^11–13^. Nevertheless, the findings of biometrics’ weights were not completely acquired under the entire consideration of biometrics.

Therefore, the purpose of this research is to establish the evaluation model of biometrics for administrators of biometrics to improve the performances. The results will exhibit the specific process of improving biometrics and the influential weights of biometrics for evaluating the performances under the full consideration of affecting criteria by adopting a hybrid MCDM model. Past research works of biometrics mainly focus on the procedures and what factors would affect biometrics and whether the effect is positive or negative. Nevertheless, decision making requires the consideration of multiple criteria with interdependence and feedback in real world. The findings for constructing decision model of biometrics make few contributions to these issues. Also, the interrelationship and influential weights among criteria of biometrics was rarely researched.

This paper uses a hybrid MCDM model including decision making trial and evaluation laboratory (DEMATEL) and DEMATEL-based analytical network process (DANP) to supplement past researches for building the decision model of biometrics. The criteria of the hybrid MCDM model for evaluating biometrics are pointed out by comprehensive literature review. The current challenges of the proposed research are experts with specialty of biometrics to develop a professional decision model, instead of customers to understand their preferences, and this empirical study is implemented in Taiwan to construct an essential pattern which can be applied to the world in the future. The DEMATEL technique implemented by the survey of experts is adopted to probe into the relationships among criteria with interdependence and feedback for constructing the influential network relation map (INRM). According to the influence values of criteria in INRM, the innovation strategies for improving biometrics can be observed. The influential weights of criteria for evaluating biometric systems can be derived to overcome the issues of criteria with dependence and feedback by utilizing DANP based on the fundamental theory of analytical network process (ANP). To identify the important criteria, the influential weights of criteria are ranked by values. In addition, the weights can be used to evaluate the performances of biometrics via simple additive weighting. Few researches use a hybrid MCDM model for improving biometrics. As a result, an empirical study of biometrics is adopted to offer administrators of biometrics with a valuable decision model to develop optimal biometric systems. The contributions of proposed research compared to the existing state-of-art research can be summarized as follows:

- The INRM of biometrics can be built.
- The interrelationships within biometrics criteria can be determined by utilized DEMATEL to construct the efficient improvement strategies.
- The influential weights for understanding the diverse importance when evaluating the performances of biometrics can be calculated via DANP.

The rest of this research is organized as following. Literature review regarding to the criteria for evaluating biometrics is presented in the next section. Subsequently, hybrid MCDM methods to build a decision model and an empirical case of biometrics are exhibited. Finally, conclusions are showed.

## Literature review for criteria of biometrics

Some of the past research are isolated, disorganized, or limited in time causing discontinuous and non-integrated attempts^8^. Therefore, it is necessary to systematically build a multiple criteria model for assessing biometric technologies. Biometric Cryptographic Keys (BCKs) can be estimated by using two main dimensions: accuracy and security^14^. Furthermore, Common Criteria (CC) and Common Evaluation Methodology (CEM) were used to evaluate information technology security in biometrics^15^. Also, usability, security, and data quality can be utilized to evaluate biometric systems^16^. There were four factors that influenced the performances of biometrics in relation to accuracy (different environmental conditions), universality (illness or disabilities), distinctiveness (variable acquisition conditions), and acceptability (spoof attacks)^17^. According to the above literature review, two main dimensions of biometrics can be concluded as: usability and security.

In the dimension of usability, the human physiological and/or behavioral characteristics should meet four criteria: universality, uniqueness, permanence, and collectability, when they are considered as biometrics^18^. Uniqueness and permanence were critical for performance evaluation of biometric systems in the seven diverse criteria: universality, uniqueness, permanence, collectability, performance, acceptance, and circumvention^19^. Moreover, universality, collectability, uniqueness, and permanence were used to evaluate Event-Related Potential (ERP) biometrics^20,21^. Besides, uniqueness and permanence played an important role when designing biometric systems, because they were the foundation of biometric recognition^6^.

As for the dimension of security, the accuracy of biometric systems can be influenced by four criteria: interaction among the sensor and the individual (e.g., change angle when fingerprints acquisition), changes of sensor attributes (e.g., optical vs. solid-state fingerprint sensor), environment condition (e.g. high humidity causing sensor being not sensitive), and variations in the biometric feature (e.g., having plastic surgery)^22^. Biometrics were evaluated for online banking and asserted that secure of online systems was very important to make sure the services and assets of customers are safe^23^. The evaluation of biometric systems’ security included two criteria: biometric systems (composed of devices and algorithms) and biometric conditions (composed of environmental and operational conditions)^16^. The performances of biometrics can be improved by the great advances in computing and storage causing the great algorithms for dealing with mass biometric data^6^. A novel biometric quality assessment (BQA) algorithm was adopted to advance the reliability of face recognition systems^24^.

The dimensions and criteria for evaluating biometrics can be summarized as follows based on the literature review. Evaluating biometrics comprise two main dimensions: usability (*D*_1_) and security (*D*_2_). The usability dimension is influenced by four criteria: universality (*C*_1_), collectability (*C*_2_), uniqueness (*C*_3_), and permanence (*C*_4_). In addition, the security dimension is affected by biometric systems (*C*_5_) and biometric conditions (*C*_6_).

## Methods including DEMATEL and DANP

To probe into the interrelationship among biometrics and the influential weights of dimensions/criteria when evaluating biometrics are the main issues of this paper. Therefore, to provide decision makers with a useful decision model to make the optimal decisions, a hybrid MCDM model is used to take multiple criteria into consideration at the same time. To construct the INRM for administrators of biometrics to design and make suitable biometric systems, the method of DEMATEL is adopted. Afterwards, the influential weights of criteria can be calculated. The proposed methods from the experts’ point of view mainly provide administrators of biometrics with an evaluation model to improve the systems, and thus the preferences of normal customers cannot be understood. Also, the additionally suitable criteria should be taken into consideration as the progress of technology to make the evaluation model more effective. The main stages can be introduced as follows.

### Constructing the INRM by DEMATEL

The DEMATEL technique is employed to explore the interdependent and feedback problems among criteria for building the INRM^25^. This method has been practically utilized to decision-making problems of various fields, including solar farms, portfolio selection, online reputation, search engine optimization (SEO), and so on^26–29^.

The method is presented as follows: first, the influence matrix is obtained by scores. The experts are required to point out the degrees of influence among criteria in questionnaire sheet; i.e., to indicate how much the criteria affect each other. The influence matrix ***A*** can thus be acquired. Second, the normalized influence matrix ***H*** can be calculated by using Eqs. (1) and (2) to normalize ***A***.

1 $$ {\varvec{H}}{ = }m \cdot {\mathbf{A}} $$

2 $$ m = \min \left\{ {\frac{1}{{\max_{i} \sum\nolimits_{j = 1}^{n} {|a_{ij} |} }},\frac{1}{{\max_{j} \sum\nolimits_{i = 1}^{n} {|a_{ij} |} }}} \right\} $$

Thirdly, the total influence matrix $${\varvec{T}}$$ can be obtained by utilizing the formula, $${\varvec{T}} = \user2{H + H}^{2} + {\varvec{H}}^{3} { + } \cdots + {\varvec{H}}^{q} { = }{\varvec{H}}{(}{\varvec{I}}{ - }{\varvec{H}}{)}^{ - 1}$$, when $$\lim_{q \to \infty } {\varvec{H}}^{q} = [0]$$$$_{n \times n} ,$$ where ***I*** denotes the identity matrix. The fourth step: definition of the INRM through the vectors ***r*** and ***d***, which are defined the sum of the rows and the sum of the columns separately within the total influence matrix $$\user2{T = }{[}t_{ij} {]}_{n \times n}$$ via the Eqs. (3) and (4) then

3 $$ {\varvec{r}} = [r_{i} ]_{n \times 1} = \left[ {\sum\limits_{j = 1}^{n} {t_{ij} } } \right]_{n \times 1} $$

4 $$ {\varvec{d}} = [d_{j} ]_{n \times 1} = \left[ {\sum\limits_{i = 1}^{n} {t_{ij} } } \right]^{\prime }_{1 \times n} $$

where the superscript $^{\prime}$ represents transpose. If $r_{i}$ stands for the row sum of the *i*th row in matrix ${\varvec{T}}$, then $r_{i}$ displays the sum of direct and indirect influences of criterion $i$ on the all other criteria. And, if $s_{j}$ represents the column sum of the *j*th column of matrix ${\varvec{T}}$, then $s_{j}$ shows the sum of direct and indirect receive the effects that criterion $j$ is received the effects from the all other criteria. Moreover, when $i = j$ the sum of the row and column aggregates $(r_{i} + d_{i} )$, it exhibits the giving and received degree of influences; i.e., $(r_{i} + d_{i} )$ presents the intensity of the important role that the *i*th criterion plays in the problem. When $(r_{i} - d_{i} )$ is positive, the *i*th criterion affects other criteria. However, if $(r_{i} - d_{i} )$ is negative, other criteria influence the *i*th criterion (i.e., *i*th criterion to be influenced from other criteria). And thus the INRM can be established for analyzing how to improve and set improvement strategies.

### Obtaining criteria’s influential weights by using the DANP

Decision makers almost consider multiple criteria and determine the relative influential weights of criteria when evaluating performance^30^. We can use the DEMATEL technique to build the interacting relationship of normalized influential matrix ${\varvec{T}}_{c}^{\alpha }$ by dimensions in criteria, then we can transpose the normalized influential matrix ${\varvec{T}}_{c}^{\alpha }$; the unweighted supermatrix ${\varvec{W}}{ = (}{\varvec{T}}_{c}^{\alpha } {)^{\prime}}$ can be obtained the most accurate weight of influence using the basic concept of ANP. We use the normalized of influential matrix ${\varvec{T}}_{D}^{\alpha }$ of dimensions as weighting with unweighted supermatrix ${\varvec{W}}$, the weighted supermatrix ${\varvec{W}}^{\alpha }$ can be obtained. Finally we can multiply by itself several times, until the supermatrix has converged and become a long-term stable supermatrix to a sufficiently large power z by $\mathop {\lim }\limits_{z \to \infty } \;({\mathbf{W}}^{\alpha } )^{z}$ to deal with problems of dependence and feedback among criteria for obtaining the influential weights of criteria. Thus, DANP (DEMATEL–based ANP) contains the following steps.

The DANP consists of four steps. In the first step the influence network structure based can be constructed on DEMATEL technique. Secondly, obtain the unweighted supermatrix. The total influence matrix $T_{{{}_{c}}}^{{}}$ displayed in Eq. (5) is derived from DEMATEL.5

Use the total degree of influence to normalize each level of *T*_C_ for obtaining ***T***_**C**_^α^ by Eq. (6).6
where ${\mathbf{T}}_{c}^{\alpha 11}$ can be calculated via Eqs. (7) and (8); by the same way ${\mathbf{T}}_{c}^{\alpha nn}$ can be acquired.

7 $$ d_{i}^{11} = \sum\limits_{j = 1}^{{m_{1} }} {t_{{_{C} ij}}^{11} } ,\;i = 1,2, \ldots ,m_{1} $$

8 $$ {\mathbf{T}}_{{{}_{C}}}^{\alpha 11} = \left[ {\begin{array}{*{20}c} {t_{{_{C} 11}}^{11} /d_{1}^{11} } & \cdots & {t_{{_{C} 1j}}^{11} /d_{1}^{11} } & \cdots & {t_{{_{C}^{{1m_{1} }} }}^{11} /d_{1}^{11} } \\ \vdots & {} & \vdots & {} & \vdots \\ {t_{{_{C} i1}}^{11} /d_{i}^{11} } & \cdots & {t_{{_{C} ij}}^{{{}_{{}}11}} /d_{i}^{11} } & \cdots & {t_{{_{C}^{{im_{1} }} }}^{{{}_{{}}11}} /d_{i}^{11} } \\ \vdots & {} & \vdots & {} & \vdots \\ {t_{{_{C}^{{m_{1} 1}} }}^{11} /d_{{m_{1} }}^{11} } & \cdots & {t_{{_{C}^{{m_{1} j}} }}^{11} /d_{{m_{1} }}^{11} } & \cdots & {t_{{_{C}^{{m_{1} m_{1} }} }}^{11} /d_{{m_{1} }}^{11} } \\ \end{array} } \right] = \left[ {\begin{array}{*{20}c} {t_{{_{C} 11}}^{\alpha 11} } & \cdots & {t_{{_{C1j} }}^{\alpha 11} } & \cdots & {t_{{_{C}^{{1m_{1} }} }}^{\alpha 11} } \\ \vdots & {} & \vdots & {} & \vdots \\ {t_{{_{C} i1}}^{\alpha 11} } & \cdots & {t_{{_{C} ij}}^{\alpha 11} } & \cdots & {t_{{_{C}^{{im_{1} }} }}^{\alpha 11} } \\ \vdots & {} & \vdots & {} & \vdots \\ {t_{{_{C}^{{m_{1} 1}} }}^{\alpha 11} } & \cdots & {t_{{_{C}^{{m_{1j} }} }}^{\alpha 11} } & \cdots & {t_{{_{C}^{{m_{1} m_{1} }} }}^{\alpha 11} } \\ \end{array} } \right] $$

The unweighted supermatrix can be obtained by utilizing the interdependent relationship in group to array ${\mathbf{T}}_{{{}_{C}}}^{\alpha }$ by Eq. (9).9
where ${\mathbf{W}}^{11}$ is displayed by Eq. (10), and ${\mathbf{W}}^{nn}$ in the same way. A blank space or 0 in the matrix presents independence of the group of criteria or a single criterion in relation to other criteria.

10 $$  W^{{11}}  = ({T}^{{11}} )^{{\prime }}  = \begin{array}{*{20}c}    {\begin{array}{*{20}c}    {c_{{11}} } &  \cdots  & {c_{{1i}} } &  \cdots  & {c_{{1m_{{_{{1}} }} }} }  \\   \end{array} }  \\    {\begin{array}{*{20}l}    {c_{{11}} } \hfill  \\     \vdots  \hfill  \\    {c_{{1j}} } \hfill  \\     \vdots  \hfill  \\    {c_{{1m_{{_{{1}} }} }} } \hfill  \\   \end{array} \left[ {\begin{array}{*{20}c}    {t_{{c{11}}}^{{\alpha {11}}} } &  \cdots  & {t_{{ci{1}}}^{{\alpha {11}}} } &  \cdots  & {t_{{cm_{{_{{1}} }} 1}}^{{\alpha {11}}} }  \\     \vdots  & {} &  \vdots  & {} &  \vdots   \\    {t_{{c1j}}^{{\alpha {11}}} } &  \cdots  & {t_{{cij}}^{{\alpha {11}}} } &  \cdots  & {t_{{cm_{{_{{1}} }} j}}^{{\alpha {11}}} }  \\     \vdots  & {} &  \vdots  & {} &  \vdots   \\    {t_{{c{1}m_{{_{{1}} }} }}^{{\alpha {11}}} } &  \ldots  & {t_{{cim_{{_{{1}} }} }}^{{\alpha {11}}} } &  \cdots  & {t_{{cm_{{_{{1}} }} m_{{_{{1}} }} }}^{{\alpha {11}}} }  \\   \end{array} } \right]}  \\   \end{array}   $$

The third step is to obtain the weighted supermatrix. The total influence matrix of dimensions ${\mathbf{T}}_{D}$ is computed by Eq. (11). Use the total degree of influence to normalize each level of ${\mathbf{T}}_{D}$ by Eq. (12) to obtain ${\mathbf{T}}_{D}^{\alpha }$.

$$d_{i} = \sum\limits_{j = 1}^{n} {t_{D}^{ij} }$$, $$i = 1,2,...,n$$

11 $$  {\mathbf{T}}_{D} {\text{ = }}\left[ {\begin{array}{*{20}c}    {t_{{_{D} }}^{{11}} } &  \cdots  & {t_{{_{D} }}^{{1j}} } &  \cdots  & {t_{{_{D} }}^{{1n}} }  \\     \vdots  & {} &  \vdots  & {} &  \vdots   \\    {t_{{_{D} }}^{{i1}} } &  \cdots  & {t_{{_{D} }}^{{ij}} } &  \cdots  & {t_{{_{D} }}^{{in}} }  \\     \vdots  & {} &  \vdots  & {} &  \vdots   \\    {t_{{_{D} }}^{{n1}} } &  \cdots  & {t_{{_{D} }}^{{nj}} } &  \cdots  & {t_{{_{D} }}^{{nn}} }  \\   \end{array} } \right] $$

12 $$ {\mathbf{T}}_{D}^{\alpha } = \left[ {\begin{array}{*{20}c} {t_{{_{D} }}^{11} /d_{1} } & \cdots & {t_{{_{D} }}^{1j} /d_{1} } & \cdots & {t_{{_{D} }}^{1n} /d_{1} } \\ \vdots & {} & \vdots & {} & \vdots \\ {t_{{_{D} }}^{i1} /d_{i} } & \cdots & {t_{{_{D} }}^{ij} /d_{i} } & \cdots & {t_{{_{D} }}^{in} /d_{i} } \\ \vdots & {} & \vdots & {} & \vdots \\ {t_{{_{D} }}^{n1} /d_{n} } & \cdots & {t_{{_{D} }}^{nj} /d_{n} } & \cdots & {t_{{_{D} }}^{nn} /d_{n} } \\ \end{array} } \right]\;\; = \left[ {\begin{array}{*{20}c} {t_{{_{D} }}^{\alpha 11} } & \cdots & {t_{{_{D} }}^{\alpha 1j} } & \cdots & {t_{{_{D} }}^{\alpha 1n} } \\ \vdots & {} & \vdots & {} & \vdots \\ {t_{{_{D} }}^{\alpha i1} } & \cdots & {t_{{_{D} }}^{\alpha ij} } & \cdots & {t_{{_{D} }}^{\alpha in} } \\ \vdots & {} & \vdots & {} & \vdots \\ {t_{{_{D} }}^{\alpha n1} } & \cdots & {t_{{_{D} }}^{\alpha nj} } & \cdots & {t_{{_{D} }}^{\alpha nn} } \\ \end{array} } \right] $$

The weighted supermatrix ${\varvec{W}}^{\alpha }$ can be derived from normalizing ${\mathbf{T}}_{D}^{\alpha }$ into the unweighted supermatrix ${\varvec{W}}$ for normalized supermatrix ${\varvec{W}}^{\alpha }$ exhibited in Eq. (13).

13 $$ {\varvec{W}}^{\alpha } = {\varvec{T}}_{D}^{\alpha } {\varvec{W}} = \left[ {\begin{array}{*{20}c} {t_{D}^{{\alpha {11}}} \times {\varvec{W}}^{{{11}}} } & \cdots & {t_{D}^{{\alpha i{1}}} \times {\varvec{W}}^{i1} } & \cdots & {t_{D}^{\alpha n1} \times {\varvec{W}}^{n1} } \\ \vdots & {} & \vdots & {} & \vdots \\ {t_{D}^{\alpha 1j} \times {\varvec{W}}^{1j} } & \cdots & {t_{D}^{\alpha ij} \times {\varvec{W}}^{ij} } & \cdots & {t_{D}^{\alpha nj} \times {\varvec{W}}^{nj} } \\ \vdots & {} & \vdots & {} & \vdots \\ {t_{D}^{\alpha 1n} \times {\varvec{W}}^{1n} } & \cdots & {t_{D}^{\alpha in} \times {\varvec{W}}^{in} } & \cdots & {t_{D}^{\alpha nn} \times {\varvec{W}}^{nn} } \\ \end{array} } \right] $$

Fourthly, obtain the influential weights of DANP. The weighted supermatrix ${\varvec{W}}^{\alpha }$ multiplies by itself many times to calculate the limit supermatrix based on the concept of Markov Chain. The influential weight of each criterion can thus be obtained by $\lim_{z \to \infty } ({\mathbf{W}}^{\alpha } )^{z}$. The influential weights of DANP are obtained by the limit supermatrix application ${\mathbf{W}}^{\alpha }$ with power *z*, an adequately large integer, until the supermatrix ${\mathbf{W}}^{\alpha }$ has converged and becomes a long-term stable supermatrix to obtain the global influential weight.

Consequently, the hybrid MCDM model combined the DEMATEL technique with basic concept of ANP (DEMATEL-based ANP, called DANP) can handle problems with interrelationship (interdependence and feedback) among dimensions/criteria in real world.

## Empirical case analysis

An empirical study is illustrated to propose innovation strategies for administrators of biometrics to design and make suitable biometric systems in this section. Group decision making is a famous method to contain various thinking and perspectives for solve real-world problems. Experts with related fields are invited to consider any possibilities by group decision making in this study. The data assembled from experts are analyzed by adopting the hybrid MCDM model, and the findings are exhibited in helpful modes for decision making.

### Data collection

The objects of questionnaire are experts with specialty of biometrics. Information required for sufficient evaluation the performances of biometrics are collected through interviews and filling in suitable questionnaires. A scale of 0, 1, 2, 3 and 4 displaying the degree from “no influence” to “very high influence” is adopted in the DEMATEL questionnaire. Experts are the objects of questionnaire, but not customers for analyzing the degree of satisfaction. Thus, the consensus of experts’ numbers will be tested by this study. The degree of consensus will be increasing, when samples of expert increase, i.e. the difference will be decreasing. The inquisition is implemented in May 2021.

### Comprehending the relationships among biometrics for constructing INRM

**Figure 1:**
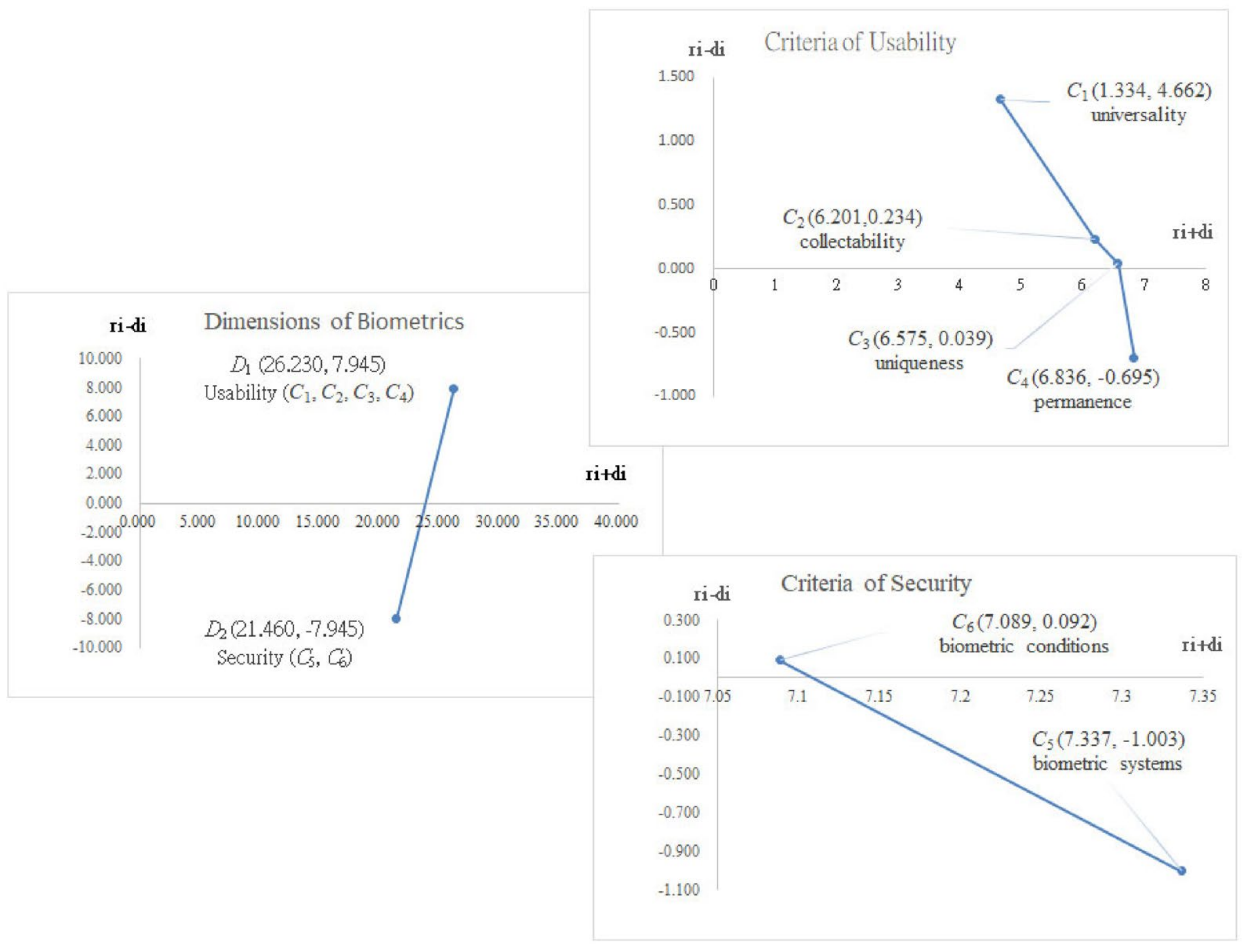
The INRM of influential relationships within biometrics.

DEMATEL technique is employed to investigate the problems of interdependence and feedback among six criteria. In the first place, the influence matrix ${\mathbf{A}}$ is presented (Table 1). Secondly, the normalized influence matrix ***H*** can be received via Eq. (1) (Table 2). At third, the total influence matrix ***T*** is obtained by using Eq. (3) (Table 3). The INRM of influential relationships within biometrics is eventually built by the vector ***r*** and vector ***d*** (Table 4) from the total influence matrix ***T*** as shown in Fig. 1.

**Table 1:** The initial influence matrix ***A***.

Criteria	*C*_1_	*C*_2_	*C*_3_	*C*_4_	*C*_5_	*C*_6_
*C*_1_	0.000	2.100	1.100	3.000	2.100	3.900
*C*_2_	1.000	0.000	3.000	3.100	4.000	2.200
*C*_3_	1.000	2.100	0.000	4.000	3.700	2.900
*C*_4_	1.000	2.900	2.900	0.000	3.800	2.000
*C*_5_	1.200	2.000	2.900	2.900	0.000	3.900
*C*_6_	2.200	3.000	3.000	3.000	3.900	0.000

**Table 2:** The normalized direct-influence matrix ***H***.

Criteria	*C*_1_	*C*_2_	*C*_3_	*C*_4_	*C*_5_	*C*_6_
*C*_1_	0.000	0.120	0.063	0.171	0.120	0.223
*C*_2_	0.057	0.000	0.171	0.177	0.229	0.126
*C*_3_	0.057	0.120	0.000	0.229	0.211	0.166
*C*_4_	0.057	0.166	0.166	0.000	0.217	0.114
*C*_5_	0.069	0.114	0.166	0.166	0.000	0.223
*C*_6_	0.126	0.171	0.171	0.171	0.223	0.000

**Table 3:** The total influence matrix ***T***.

Criteria	*C*_1_	*C*_2_	*C*_3_	*C*_4_	*C*_5_	*C*_6_
*C*_1_	0.209	0.478	0.467	0.607	0.622	0.615
*C*_2_	0.273	0.394	0.585	0.649	0.744	0.573
*C*_3_	0.281	0.514	0.451	0.700	0.747	0.614
*C*_4_	0.263	0.518	0.561	0.476	0.710	0.543
*C*_5_	0.283	0.494	0.573	0.633	0.547	0.637
*C*_6_	0.355	0.586	0.632	0.702	0.800	0.516

**Table 4:** The sum of influences giving and received.

Dimensions/criteria	*r*_*i*_	*d*_*i*_	*r*_*i*_ + *d*_*i*_	*r*_*i*_*- d*_*i*_
**Usability (*D***_**1**_**)**	**17.088**	**9.143**	**26.230**	**7.945**
Uuniversality (*C*_1_)	2.998	1.664	4.662	1.334
Collectability (*C*_2_)	3.217	2.984	6.201	0.234
Uniqueness (*C*_3_)	3.307	3.268	6.575	0.039
Permanence (*C*_4_)	3.071	3.766	6.836	-0.695
**Security (*D***_**2**_**)**	**6.758**	**14.703**	**21.460**	**-7.945**
Biometric systems (*C*_5_)	3.167	4.170	7.337	-1.003
Biometric conditions (*C*_6_)	3.590	3.499	7.089	0.092

### Finding influential weights of criteria by DANP

DANP is employed by this research to receive the level of influential weights (global weights) of six criteria shown as Table 5 ~ 7 based on the construction of the influence network from DEMATEL. The empirical findings discover that experts put more attention on biometric systems (*C*_5_), biometric conditions (*C*_6_), and permanence (*C*_4_); however, less on collectability (*C*_2_) and universality (*C*_1_). The results reveal that the level of influential weights is much higher in biometric systems, biometric conditions, and permanence. More specifically, biometric systems (*C*_5_) gets the highest influential weight of 0.369, followed by biometric conditions (0.32) and permanence (0.098). When comparing criteria among dimension, experts consider permanence as the most important criterion in the dimension of usability (*D*_1_). biometric systems is regarded by experts as the most important criterion in the dimension of security (*D*_2_). Received results (ranked top one) present that biometric systems (*C*_5_) is the last criterion which can be neglected when improving biometrics.

**Table 5:** The unweighted supermatrix $W = {(}{\varvec{T}}_{c}^{\alpha } )^{\prime}$.

Criteria	*C*_1_	*C*_2_	*C*_3_	*C*_4_	*C*_5_	*C*_6_
*C*_1_	0.118	0.144	0.144	0.145	0.143	0.156
*C*_2_	0.272	0.207	0.264	0.285	0.249	0.258
*C*_3_	0.265	0.308	0.232	0.309	0.289	0.278
*C*_4_	0.345	0.341	0.360	0.262	0.319	0.308
*C*_5_	0.503	0.565	0.549	0.566	0.462	0.608
*C*_6_	0.497	0.435	0.451	0.434	0.538	0.392

**Table 6:** The weighted supermatrix ${\varvec{W}}^{\alpha } = {\varvec{T}}_{D}^{\alpha } {\varvec{W}}$.

Criteria	*C*_1_	*C*_2_	*C*_3_	*C*_4_	*C*_5_	*C*_6_
*C*_1_	0.051	0.062	0.063	0.063	0.036	0.040
*C*_2_	0.118	0.090	0.115	0.124	0.063	0.066
*C*_3_	0.115	0.134	0.101	0.134	0.073	0.071
*C*_4_	0.150	0.148	0.156	0.114	0.081	0.078
*C*_5_	0.284	0.319	0.310	0.320	0.345	0.453
*C*_6_	0.281	0.246	0.255	0.245	0.401	0.292

**Table 7:** The stable matrix of DANP when $\mathop {\lim }\limits_{z \to \infty } ({\mathbf{W}}^{\alpha } )^{z}$.

Criteria	*C*_1_	*C*_2_	*C*_3_	*C*_4_	*C*_5_	*C*_6_
*C*_1_	0.046	0.046	0.046	0.046	0.046	0.046
*C*_2_	0.079	0.079	0.079	0.079	0.079	0.079
*C*_3_	0.088	0.088	0.088	0.088	0.088	0.088
*C*_4_	0.098	0.098	0.098	0.098	0.098	0.098
*C*_5_	0.369	0.369	0.369	0.369	0.369	0.369
*C*_6_	0.320	0.320	0.320	0.320	0.320	0.320

### Implications and discussions

Discussions of empirical results and innovation strategies for improving biometrics are presented as follows. In the first place, the influential relationships within biometrics suggest what administrators of biometrics should improve first is usability (*D*_1_) to enhance the performance of biometrics based on INRM established by DEMATEL. With the highest influencing value, universality (*C*_1_) especially should be greatly addressed, because it is of systematical importance to affect all other criteria. Universality is that we can find our chosen biometric characteristic in most of people. Even though we choose a common characteristic, taking a fingerprint for instance, we should consider that some people may not have an index finger and be ready to make up for that.

Secondly, the most important criterion found by DANP when improving biometrics is biometric systems (*C*_5_) whose influential weight equals 0.369. A multimodal biometrics security system of hybrid algorithms including Gaussian Mixture Models (GMMs), Artificial Neural Networks (ANNs), Fuzzy Expert Systems (FESs), and Support Vector Machines (SVMs) is demonstrated to have higher recognition rates and lower false alarms compared to unimodal biometric security systems^31^.

The proposed hybrid MCDM model combined with biometrics can be applied by administrators of biometrics in worldwide. They can adjust the influential weights of the six criteria or add more factors according to the biometrics situations of various countries to obtain useful information for decision making when improving biometrics. Furthermore, administrators of biometrics can evaluate and improve biometrics by utilizing this hybrid MCDM model. Last but not least, the state-of-the-art evaluation model uses the influential weights derived by the proposed model to find out the gaps of criteria under different biometrics’ systems for improving their performances to achieve the aspiration level by using VIse Kriterijumska Optimizacija I Kompromisno Resenje (VIKOR), which is an important tool for administrators of biometrics.

## Conclusions and remarks

The two dimensions, usability and security, are considered as a significant instrument for measuring biometrics. It has been well-known for years that the two dimensions influence biometrics. It is still unclear, nevertheless, how the evaluation criteria influence the two dimensions. The influential weights of criteria are seldom explored, although the comprehending of the importance of the criteria can be useful for administrators of biometrics to design and make suitable biometric systems.

The criteria of biometrics are showed having interrelations and self-feedback relationships by DEMATEL. Moreover, the influence levels of the six criteria are obtained via DANP. Empirical results reveal that biometric systems is the most important criterion, followed by biometric conditions, permanence, uniqueness, collectability, and universality. Experts recommend that administrators of biometrics should put more stress on biometric systems, though they must take entire criteria into consideration when making decisions of improving biometrics.

Past researches take less notice of introducing biometrics and identifying the criteria that affect it. Besides, only few preceding study attempts are concerned about the interrelationship among criteria, and the weights of criteria. Consequently, this research utilizes a hybrid MCDM model and investigates the standpoints of experts for probing into these issues. The combination of past theoretical researches and opinions of professional experts makes biometrics scales become a more useful tool, which is not offered by previous study attempts.

This research focuses on constructing the evaluation model for administrators of biometrics to improve their systems; therefore, future works are recommended to use the proposed model for comparing the performances among different biometrics systems. Moreover, further researches can add suitable criteria to explore the preferences of common customers for making the study of biometrics more complete.
